# 3D Spheroid Formation Using BMP-Loaded Microparticles Enhances Odontoblastic Differentiation of Human Dental Pulp Stem Cells

**DOI:** 10.1155/2021/9326298

**Published:** 2021-08-23

**Authors:** Tae-Jun Min, Min Ji Kim, Kyung-Jung Kang, Yeoung Jo Jeoung, Se Heang Oh, Young-Joo Jang

**Affiliations:** ^1^Department of Nanobiomedical Science & BK21 FOUR NBM Global Research Center for Regenerative Medicine, Dankook University, Cheonan 31116, Republic of Korea; ^2^Laboratory of Oral Biochemistry, College of Dentistry, Dankook University, Cheonan 31116, Republic of Korea

## Abstract

Human dental pulp stem cells (hDPSCs) are the primary cells responsible for dentin regeneration. Typically, in order to allow for odontoblastic differentiation, hDPSCs are cultured over weeks with differentiation-inducing factors in a typical monolayered culture. However, monolayered cultures have significant drawbacks including inconsistent differentiation efficiency, require a higher BMP concentration than should be necessary, and require periodic treatment with BMPs for weeks to see results. To solve these problems, we developed a 3D-cell spheroid culture system for odontoblastic differentiation using microparticles with leaf-stacked structure (LSS), which allow for the sustained release of BMPs and adequate supply of oxygen in cell spheroids. BMPs were continuously released and maintained an effective concentration over 37 days. hDPSCs in the spheroid maintained their viability for 5 weeks, and the odontoblastic differentiation efficiency was increased significantly compared to monolayered cells. Finally, dentin-related features were detected in the spheroids containing BMPs-loaded microparticles after 5 weeks, suggesting that these hDPSC-LSS spheroids might be useful for dentin tissue regeneration.

## 1. Introduction

In a normal biological system, stem cells undergo intensive cell-cell contacts to develop tissues with three-dimensional (3D) structures. This is mediated by the microenvironment including the extracellular matrix surrounding cells, which are distinct physiological cues for cell differentiation and proliferation. Two-dimensional (2D) monolayer cultures inadequately reproduce the native microenvironment of stem cells created by intrinsic and extrinsic cellular communications, resulting in an unnatural spatial distribution of signaling molecules, oxygen, and nutrients [1]. Moreover, the normal physiological behaviors of stem cells can be distorted in monolayer cultures, leading to the loss of the multilineage potential and replicative ability [2, 3]. To mimic the physiological environment, various methods have been reported for culturing cells in a 3D structure, including scaffold-free cultures [4, 5], cultures grown on various scaffold materials [6–8], and cultures embedded in gel materials [9, 10]. 3D cell aggregates, known as spheroids, yield a multicellular mass that mimics the natural cell niche [11]. Scaffold-free 3D culture systems have advantages in preventing the inflammation and infection caused by scaffold material degradation [12] and disadvantages of poor guidance on cells required for cell proliferation and differentiation. Cell spheroids have been utilized in various tissue engineering [13–15] and stem cell applications [10, 16–19]. However, only recently have cell spheroids been investigated for dental tissue regeneration, leaving much data on the topic still to be discovered. Spheroid culturing of human periodontal ligament stem cells (hPDLSCs) significantly enhanced stemness and osteogenic potential compared with hPDLSCs cultured in a monolayer [11, 20]. In addition, osteo/odontoblastic gene expression and mineralization are upregulated in immortalized mouse dental papilla cells cultured as 3D spheroids when compared with 2D monolayer-cultured cells [21]. In addition to the 3D aspects of the cell culture system, the sustained supply of cytokines or growth factors also plays an important role in mimicking the natural microenvironment and achieving effective differentiation. Biomaterial-based delivery systems in culture have been previously reported to form nanoparticles, microparticles, hydrogels, and tissue engineering scaffolds, improving the pharmacokinetics of retained factors and reducing toxicity [13–15, 22]. Recently, 3D cell culture models, including cell spheroids, have been assessed in terms of drug delivery efficacy [5–7]. In previous studies, signaling pathways stimulated by Wnt proteins, transforming growth factor-*β* (TGF*β*)/bone morphogenetic proteins (BMPs), and fibroblast growth factor (FGF) were identified to regulate tooth development [23, 24]; however, the process of dentin formation is not fully understood. Recently, we reported that the combinational treatment of BMP-2 and BMP-4 can maximize the odontoblastic differentiation efficiency of hDPSCs [25]. The induction of odontoblastic differentiation in 3D spheroid cultures via the direct addition of differentiation-inducing factors to the culture medium can be complicated compared to monolayer cultures. In monolayered cultures, oxygen and nutrition are uniformly provided to all cells, whereas in 3D spheroid cultures, the concentrations of oxygen and nutrients can be quite low at the interior of spheroids as limited by diffusion [12, 26]. Our aim was to achieve high efficiency and successful odontoblastic differentiation in a 3D culture system. For this, we established an hDPSC-spheroid complex with accompanying LSS microparticles which allowed for the sustained supply of signaling factors and improved cell adhesiveness. These hDPSCs-spheroid complexes mimicked the natural microenvironment better than conventional monolayered cultures, and even larger-scale structures showed long-lasting differentiation efficiency, more so than any other structures developed thus far (e.g., solid porous matrix- or hydrogel-based scaffold systems and conventional cell only spheroid systems).

## 2. Materials and Methods

### 2.1. Cell Culture

Human dental pulp stem cells were obtained from the dental pulp tissues extracted from wisdom teeth under guidelines approved by the IRB of the Dankook University (DKU-NON2019-004). The dental pulp tissues were collected after removing the tooth crown. The tissues were chopped and treated with 3 mg/ml collagenase type-I (Millipore) and 4 mg/ml dispase (Sigma-Aldrich) for 1 h at 37°C. Single-cell suspension was incubated in alpha-modified minimum essential medium (*α*-MEM, Hyclone) containing 20% fetal bovine serum (Hyclone) and antibiotics (Lonza) at 37°C in 5% CO_2_. For induction of odontoblastic differentiation in 2D monolayer culture, hDPSCs were treated with BMP-2 and BMP-4 as indicated concentrations in every 3 days. Stemness and differentiation potentials of independently isolated hDPSCs from patients were verified by expression of the representative mesenchymal stem cell markers and odontoblastic markers (Figure S1 and S2). Each cell batch was not combined and used separately in experiments.

### 2.2. Preparation of Microparticles with BMPs and the Release Behavior Analysis

Microparticles with leaf-stacked structure (LSS) were fabricated by a heating and cooling method as described in our previous studies [27]. In brief, polycaprolactone (PCL, 81 kDa, Lakeshore Biomaterials) was dissolved in tetraglycol (Sigma-Aldrich) in 15% at 90°C, and the solution was stored at 4°C to obtain LSS microparticles. LSS particles in the size range of 25~53 *μ*m were collected using standard testing sieves. For morphological observation of LSS microparticles, a scanning electron microscope (S-4300, Hitachi) was used. For immobilization of BMP-2 and BMP-4 in LSS microparticles, a mixture of 1 ml LSS particles and 1 ml BMP solution (1 *μ*g/ml BMP in PBS supplemented with 1% bovine serum albumin) was stored at 4°C for 3 h under positive pressure. Excess BMP solutions were removed and freeze-dried. To investigate the release behavior of BMPs from LSS microparticles, 5 mg BMPs-loaded LSS microparticles were incubated in 1 ml PBS at 37°C, and supernatant was collected every 24 h. The amount of BMPs released from LSS microparticles was detected by the ELISA kit (Duoset, R&D Systems).

### 2.3. Spheroid Formation

To prepare hDPSC-spheroids containing BMPs-loaded LSS microparticle, suspension of cell (2.7 × 10^6^ cells) and microparticle (0.3 × 10^6^ particles) was seeded onto agarose mold with concave microstructure (800 *μ*m in diameter and 400 *μ*m in depth). A hemocytometer was used to count cells and microparticles. 144 concaves per experiment were prepared. The spheroids were incubated in a growth medium for 1 week and, then, incubated for additional 4 weeks to induce odontoblastic differentiation. Size and morphological changes of spheroids were observed using a light microscope (CKX41, Olympus) and a scanning electron microscope (S-4300, Hitachi).

### 2.4. Immunocytochemistry and Immunohistochemistry

For histological observation, cell spheroids were fixed with 4% paraformaldehyde for 20 min at 4°C. After freezing at -70°C, the specimens were cut into sections of 8-*μ*m thickness and, then, stained with Hematoxylin and Eosin (H&E) staining for observation by a light microscope (CKX41, Olympus). To visualize protein expression, immunocytochemical (ICC) staining was conducted on cell spheroids. A slice cut from spheroid was washed with Tris-Buffered Saline (TBS), permeabilized with 0.1% Triton X-100 for 5 min, and blocked with 10% normal serum (in TBS containing 1% BSA) for 2 h, consecutively. Specimens were incubated with anti-BSP (ab125227, Abcam) and anti-DSPP antibodies (Santa Cruz, sc-73642) at 4°C for 12 h, followed by incubation with Alexa Fluor® 488 (Abcam) as secondary antibody for 1 h. Cell nuclei were stained with 4′,6-diamidino-2-phenylindole (DAPI, Vector Laboratories) and observed using a fluorescence microscope (Eclipse Ts2R, Nikon).

### 2.5. Quantification of Calcium Contents

For quantification of mineralization, cells or spheroids were collected and incubated in 0.6 N HCl for 24 h for decalcification. Calcium amounts were quantified using Calcium Colorimetric Assay Kit (Sigma-Aldrich). A chromogenic complex was formed between calcium ions and O-cresolphthalein, and its absorbance intensity was measured at 575 nm. The calcium amounts were calculated according to the absorbance.

### 2.6. Real-Time Polymerase Chain Reaction

Total RNA was extracted from cells or spheroids using Easy-Spin Kit (Intron), and cDNA was synthesized using ReverTra Ace qPCR RT Mix (Toyobo). The qRT-PCR was conducted with iTaq Universal SYBR Green Supermix (Bio-Rad) using specific primers (Table 1). qPCR amplifying condition consisted of 1 cycle for 30 sec at 95°C, and 40 cycles for 15 sec at 95°C of denaturation and 60 sec at 55-60°C of annealing/extension. A melt curve was constructed in the range of 65°C to 95°C with 0.5°C increments per step. The relative comparison of each gene was analyzed, and glyceraldehyde 3-phosphate dehydrogenase (GAPDH) was used for normalizing gene expression as an internal control. Three samples were analyzed for each target gene.

### 2.7. Statistical Analysis

The differences between two samples were evaluated by the *t*-test as a statistical method. A *P* value of less than 0.05 was considered statistically significant.

## 3. Results

### 3.1. Development of BMPs-Loaded Microparticles for Sustainable Delivery

**Figure 1:**
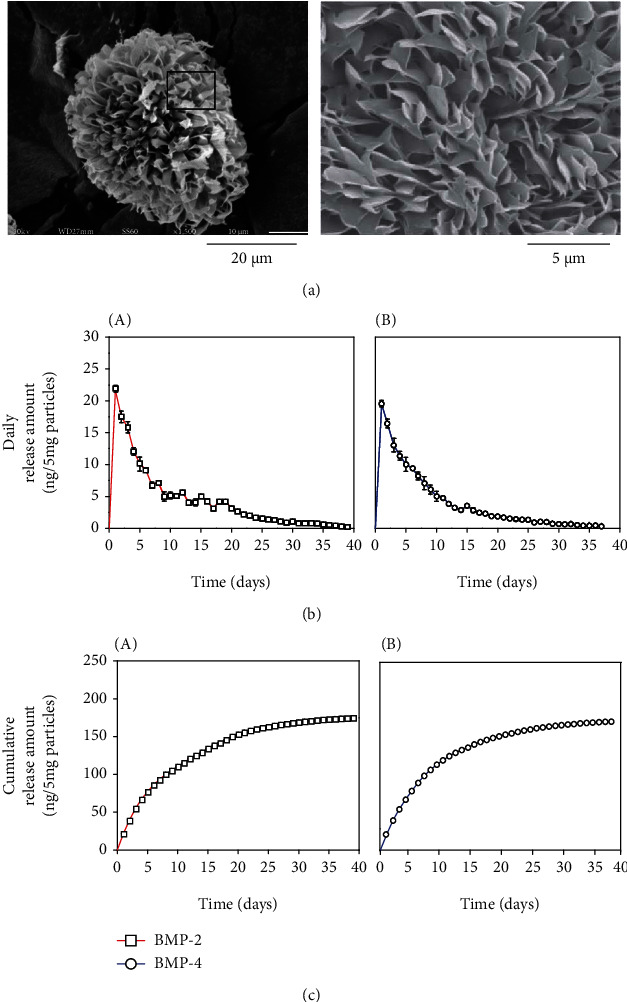
Sustainable release of BMP-2 and BMP-4 by using the microparticles of the entire leaf-stacked structure (LSS). (a) Scanning electron microscopic structure of the surface of an ELSS microparticle. (b) Daily release amounts of BMP-2 (A) and BMP-4 (B). The release behavior was analyzed daily using 5 mg of particles and continued until 0.2 ng of each BMP was released. The releases of BMP-2 and BMP-4 were analyzed for 39 days and 37 days, respectively. (c) Cumulative release amounts of BMP-2 (A) and BMP-4 (B). Over 39 days and 37 days, the cumulative release amounts of BMP-2 and BMP-4 per 5 mg of particles were 174.93 ng and 194.26 ng, respectively.

Microparticles with a leaf-stacked structure (LSS) were developed to provide an appropriate microenvironment with a continuous supply of factors and sufficient surface for cell adhesion [27]. Diameters of these particles were 25~53 *μ*m. SEM imaging (Figure 1(a)) revealed that the surfaces of these particles were covered with a laminated structure. Such laminated porous structures not only can offer a sustained release of bioactive molecules but also can improve cell adhesion and permeation of oxygen and nutrients.

To induce odontoblastic differentiation of monolayered hDPSCs, cultures were cotreated with BMP-2 and BMP-4 [25]. To facilitate odontoblastic differentiation in a 3D culture system, LSS microparticles loaded with either BMP-2 or BMP-4 were prepared. When 5 mg of microparticles was resuspended in 10 *μ*g/ml each BMP solution, maximal loading amounts of BMP-2 and BMP-4 were 206.16 ng/5 mg particles and 194.26 ng/5 mg particles, respectively. After a high initial release of factors between day 1 and day 7, the total duration of release above the effective concentration of 0.2 ng/ml was 39 days in case of BMP-2 and 37 days in case of BMP-4 (Figure 1(b), A and B). Based on the release behavior, total accumulated amounts of released BMP-2 and BMP-4 were estimated to be 174.93 ng/5 mg particles and 150.64 ng/5 mg particles, respectively (Figure 1(c), A and B). To sum up, 84.85% and 77.55% of total loading amounts of BMP-2 and BMP-4 were released over 37 and 39 days, respectively. These data suggested that BMPs were continuously released from LSS microparticles for at least 37 days, allowing them to achieve continuous differentiation induction without an intermittent stimulation.

### 3.2. Formation of hDPSC Spheroids with BMP-Loaded LSS Microparticles

**Figure 2:**
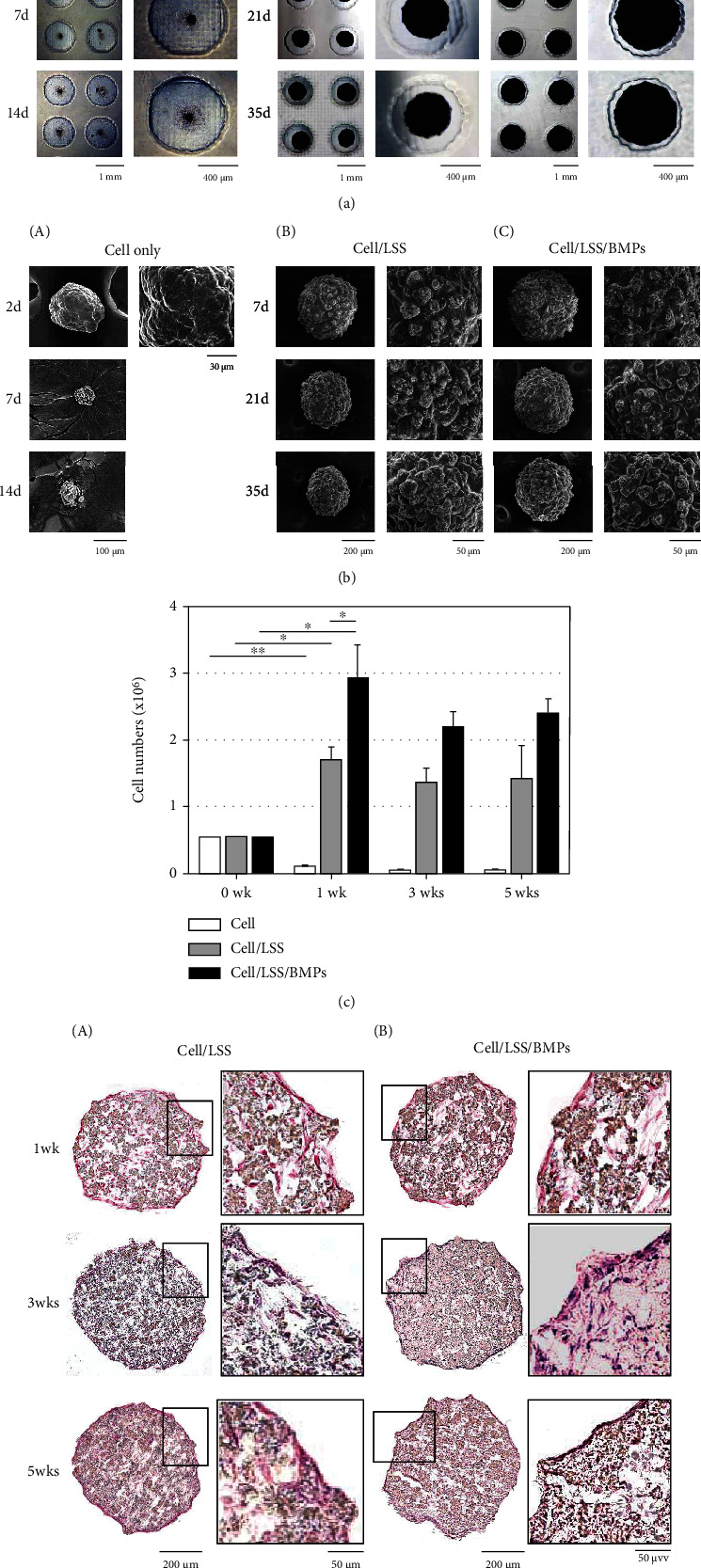
Formation of hDPSC spheroids containing the BMPs-loaded LSS microparticles. (a, b) Photographic and scanning electron microscopic image of hDPSC spheroids. (A) hDPSC spheroid without particles. (B) hDPSC spheroid with particles. (C) hDPSC spheroid with BMPs-loaded particles. (c) Cell proliferation of hDPSCs in spheroids containing microparticles after culturing for 1, 3, and 5 weeks. 0 wk indicated the cell numbers added for spheroid formation. White bars, cell numbers in spheroids containing particles without BMPs; black bars, cell numbers in spheroids containing particles loaded BMPs. ^∗^*P* < 0.05; ^∗∗^*P* < 0.01; ^∗∗∗^*P* < 0.001; NS: not significant. (d) H&E staining of paraffin section of hDPSC-spheroids containing LSS particles. As well as covering on spheroid by cell layers, cells were detected inside of spheroid. (A) hDPSC spheroid with particles. (B) hDPSC spheroid with BMPs-loaded particles.

To construct cell aggregates, 2.7 × 10^6^ hDPSCs and 0.3 × 10^6^ BMPs-loaded LSS particles were mixed in an agarose concave microwell. In our previous report [25], monolayered hDPSCs required treatment with both 33.3 ng/ml BMP-2 and 3.3 ng/ml BMP-4 per day to produce odontoblastic differentiation. To induce odontoblastic differentiation under conditions similar to a two-dimensional culture, 3.2 mg BMP-2-loaded LSS particles (0.270 × 10^6^ particles) and 0.32 mg BMP-4-loaded LSS particles (0.027 × 10^6^ particles) were used for spheroid formation. These particles released either 1.17 ng/ml BMP-2 or 0.12 ng/ml BMP-4 per day. One spheroid body was constructed per well (Figures 2(a) and 2(b)), resulting in a total of 144 cell spheroids. Cell spheroids without LSS particles decreased in size over time. They almost disappeared after five weeks (Figure 2, A in (a) and (b)). This phenomenon might be due to cell death as cell necrosis in the central region of spheroids has been occasionally observed in larger spheroid structures [28]. However, cell spheroids with LSS microparticles (Cell/LSS and Cell/LSS/BMPs) did not shrink much during incubation (Figure 2, A and C in (a) and (b)). To analyze cell viability, an MTT assay was performed with 30 spheroids containing LSS particles. By the first week, cells in spheroids without particles sharply decreased in number, while cells in Cell/LSS and Cell/LSS/BMPs proliferated and cell numbers increased (Figure 2(c)). Over a period of longer than three weeks, the number of cells remained somewhat unchanged except for spheroids with only cells which showed decreased number over the same period (Figure 2(c), bars in 3-5 wks). Therefore, mixed hDPSCs and LSS particle spheroid system was appropriate for a long-term differentiation of hDPSCs. Hematoxylin and eosin (H&E) staining revealed cells in the core region of spheroids due to a high survival rate. It also revealed that the gap between cells and particles narrowed as the incubation time increased (Figure 2(d)). In both LSS particles and BMPs-loaded LSS particles, cells either wrapped close to particles or hung between particles.

### 3.3. Enhancement of Odontoblastic Differentiation in hDPSC/LSS Spheroids Releasing BMP-2 and BMP-4

**Figure 3:**
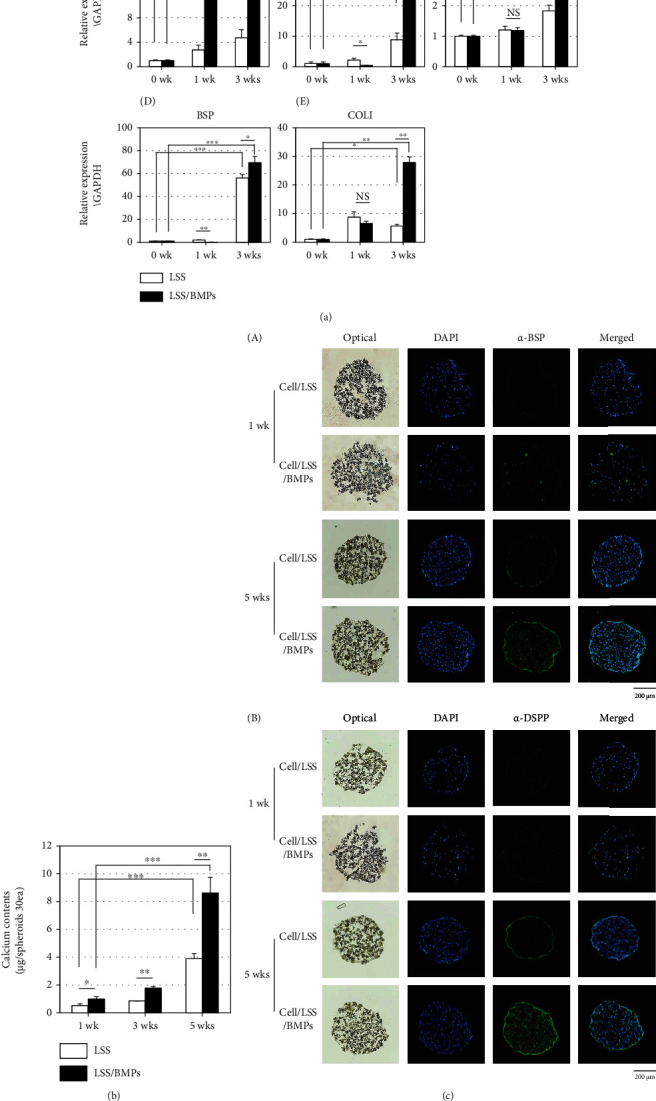
Odonto/osteoblastic differentiation of 3D spheroid-cultured hDPSCs improves by the sustainably released BMP-2 and BMP-4. (a) Odonto/osteoblastic marker gene expression of hDPSC spheroids. (A) Osterix. (B) Dentin matrix protein-1. (C) Osteocalcin. (D) Bone sialoprotein. (E) Collagen type-1. ^∗^*P* < 0.05; ^∗∗^*P* < 0.01; ^∗∗∗^*P* < 0.001; NS: not significant. (b) Calcium deposition in spheroids. 30 spheroids were collected to analyze the amounts of calcium as followed in Experimental procedures. White bars, cell numbers in spheroids containing particles without BMPs; black bars, cell numbers in spheroids containing particles loaded BMPs. ^∗^*P* < 0.05; ^∗∗^*P* < 0.01; ^∗∗∗^*P* < 0.001. (c) Immunohistochemical staining of hDPSCs spheroids for two representative odontoblastic markers such as bone sialoprotein (A) and dentin sialophosphoprotein (B). The paraffin section of hDPSC-spheroids collected at an indicated time was treated with the primary antibody, followed by FTIC-conjugated secondary antibody. LSS: spheroids containing particles without BMPs; LSS/BMPs: spheroids containing particles loaded BMPs.

Odonto/osteoblastic differentiation of hDPSCs cultured in spheroids was evaluated based on the transcriptional expression of odonto/osteogenic markers. Overall, gene expression levels of representative markers were increased in spheroids even without BMP-2 or BMP-4 (Figure 3(a), LSS in a-f), although their expression levels were increased more in spheroids with BMPs-loaded LSS particles (Figure 3(a), LSS/BMPs in a-f). After 3 weeks of differentiation, expression levels of osterix (OSX) and dentin matrix protein-1 (DMP-1) were significantly increased in spheroids with BMPs-loaded LSS particles than in spheroids without LSS particles (Figure 3(a), A and B). Expressions levels of osteocalcin (OCN), bone morphogenetic protein (BSP), and type I collagen (COLI) in spheroids with BMPs-loaded particles were also increased than in those without BMPs (Figure 3(a), C–E). Calcium deposition is known to be an important indicator of odonto/osteoblastic differentiation. Thus, calcium content was quantified for 30 spheroids. It was found that spheroids containing both LSS and LSS/BMPs particles showed a significant time-dependent increase in calcium deposition (Figure 3(b)). However, the amount of calcium accumulated in spheroids with ELSS/BMPs was more than doubled compared to that in spheroids without BMPs over five weeks (Figure 3(b), bars in 5wks). In addition, the immunohistochemical analysis clearly showed that BSP and DSPP as two representative odonto/osteogenic markers were strongly increased in spheroids with LSS/BMPs after 5 weeks (Figure 3(c), A and B).

### 3.4. Odontoblastic Differentiation of hDPSCs in Monolayered Cultures and LSS/Cell Spheroid Cultures

**Figure 4:**
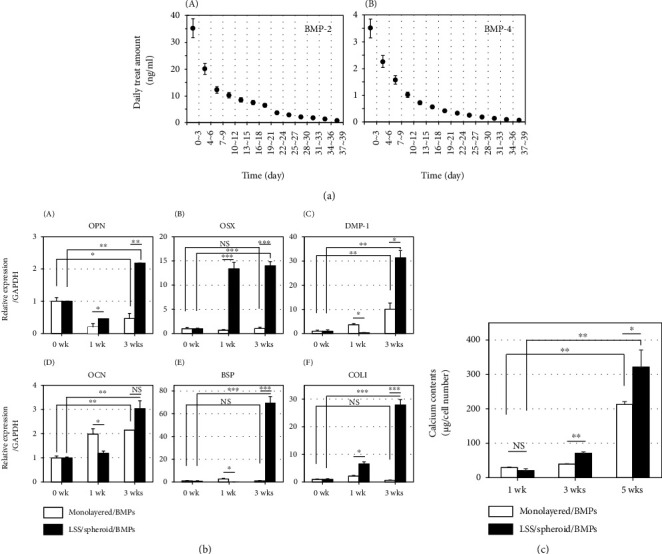
Comparison of odonto/osteoblastic differentiation of 2D monolayer-cultured hDPSCs and 3D spheroid-cultured hDPSCs. (a) Daily treat amounts of BMP-2 (A) and BMP-4 (B) in 2D monolayered culture. hDPSCs on culture dish were treated with BMPs once every 3 days. Based on the release behavior from LSS particles (Figure 1(b)), the daily average value was calculated from the sum of three days' values and displayed on the graph as a point. (b) Odonto/osteoblastic marker gene expression in hDPSCs cultured in 2D monolayer and in 3D spheroids. (A) Osteopontin. (B) Osterix. (C) Dentin matrix protein-1. (D) Osteocalcin. (E) Bone sialoprotein. (F) Collagen type-1. ^∗^*P* < 0.05; ^∗∗^*P* < 0.01; ^∗∗∗^*P* < 0.001; NS: not significant. (c) Calcium deposition in hDPSCs cultured in 2D monolayer and in 3D spheroids. 30 spheroids were collected to analyze the amounts of calcium as followed in Experimental procedures. White bars, 2D monolayer culture treated with BMPs; black bars, 3D spheroids containing particles loaded BMPs. ^∗^*P* < 0.05; ^∗∗^*P* < 0.01; NS: not significant.

In traditional monolayered cultures, BMP-2 and BMP-4 are added to hDPSCs once every two or three days to facilitate odonto/osteoblastic differentiation [25]. To compare differentiation efficacies of hDPSCs cultured in 2D monolayers and 3D spheroid systems, monolayered cells were treated with these two BMPs at concentrations approximately equal to the concentration of BMPs released from LSS microparticles. Average amounts of BMP-2 and BMP-4 added into the 2D culture during the first three days at a concentration ratio of 10 : 1 were 33.3 ng/ml and 3.3 ng/ml, respectively. After that, monolayered cultures were treated with BMPs once every three days for five weeks in a manner mimicking the release behavior from 3D spheroids (Figure 4(a)). Compared to spheroid cultures, the amount of BMPs required in 2D cultures was doubled. However, they were treated to decrease at the same ratio with releasing behavior of LSS particles. When expression levels of odonto/osteogenic markers were evaluated, all markers were significantly increased in 3D spheroid-cultured hDPSCs after 3 weeks of differentiation than in monolayered cells (Figure 4(b)). In addition, based on the same number of cells, the calcium content in 3D spheroids was increased 1.5 times compared to that in monolayered cultures (Figure 4(c)), suggesting a higher differentiation efficiency could be achieved with our LSS/hDPSCs spheroid system using vastly smaller amounts of cytokines than in traditional monolayered cultures.

## 4. Discussion

3D spheroid cultures are excellent for enhancing cell-cell and cell-extracellular matrix (ECM) interactions, mimicking the natural environment of tissues. In this study, we present an exciting spheroid culture system combined with a drug delivery system for the odonto/osteoblastic differentiation of hDPSCs. Without additional treatment with differentiation factors, hDPSCs do not differentiate well into odontoblasts or osteoblasts in traditional monolayered cultures [25]. On the other hand, it was recently reported that 3D culture systems can induce differentiation without additional inducing factors in both spheroids and scaffold-embedded cultures [11, 20, 21, 26, 29]. Nevertheless, because growth factors and cytokines are important for modulating cell proliferation, differentiation, and cell adhesion, the addition of inducers is still essential for proper differentiation. However, growth factors and cytokines only act locally and possess short half-lives and short diffusion distances through ECMs. For example, FGF and PDGF have a half-life of only 3 minutes and less than 2 minutes, respectively, due to enzymatic degradation and inactivation in physiological culture conditions [15]. Therefore, 3D cultures should be constructed in conjunction with additional drug delivery systems to sustainably release adequate growth factors and cytokines. The microparticles with the leaf-stacked structure (LSS) used in this study were suitable for cell attachment as well as the sustainable release of differentiation-inducing factors. In our recent study, we demonstrated that the microparticles with the leaf-stacked structure (LSS) significantly prevent cell necrosis in human bone marrow-derived mesenchymal stem cells (hBMSCs) (data not shown). Therefore, we can assume that cell viability in hDPSCs-spheroids with LSS particles was greatly improved in this experiment.

As shown in Figure 2, the cells in spheroid structures with LSS particles had increased viability compared to cell aggregates formed without LSS particles, and the spheroid size did not shrink even after 5 weeks of incubation. The findings were very surprising in light of the fact that more than a week or two of cell differentiation experiments were difficult when spheroids made from simple lumps of cells were used in previous studies [20, 21].

Previously, we determined the optimal concentration of BMP-2 and BMP-4 to maximize the efficiency of odontoblastic differentiation of hDPSCs: 33.2 ng/ml BMP-2 and 3.3 ng/ml BMP-4 per day [25]. Because the concentrations of BMP-2 and BMP-4 have treated at 10 : 1, two types of LSS particles loaded with BMP-2 or BMP-4 were mixed in a 10 : 1 ratio to meet proper differentiation conditions. The biggest advantage of the LSS/hDPSCs spheroid system was that it could be applied to experiments by counting the number of spheroids directly. Other studies that may reflect dual-delivery systems have recently been published. A novel drug-delivery system based on double-layered microspheres differentially released SDF-1 and BMP-2 [30]. The mesoporous silica nanoparticles-embedded core-shell nanofiber membrane provides for an effective delivery of dual drugs [31]. Together with our results, these reports will provide useful information for guided tissue regeneration.

Although the amounts of BMPs released from LSS microparticles were much less than those required in 2D monolayered cultures, the odonto/osteoblastic differentiation and mineral formation in LSS/cell spheroid-cultured hDPSCs were significantly increased after 3-5 weeks of culture compared to the monolayered but more heavily treated cells (Figure 3). These results suggest that the differentiation efficiency of hDPSCs can be enhanced in this 3D spheroid culture system using much smaller amounts of cytokines compared to 2D cultures. Previously, Chen et al. reported that periodontal ligament fibroblasts in scaffolds containing a mixture of BMP-2 and IGF-1 showed a high osteoblastic differentiation efficiency [26]. As one step further from the previous research, our dual-microparticle containing spheroids can be used to release multiple growth factors or cytokines in a controlled and independent fashion, thereby representing a promising 3D culture system for tissue engineering. In the future, multiple cell spheroids constructed with different cell types and LSS microparticles loaded with various factors could serve as building blocks in complex tissue engineering applications.

## 5. Conclusions

We established a 3D-spheroid culture system of hDPSCs for odontogenic differentiation. In this spheroid system, the microparticles with leaf-stacked structure (LSS) were used. LSS-microparticles allowed sustained release of BMPs and oxygen supply for 40 days. Odontogenic differentiation and mineralization increase significantly compared to monolayered cells, suggesting that these large-scale hDPSCs-LSS spheroids containing long-lasting viability might be useful for dentin regeneration.

## Figures and Tables

**Table 1:** Primer sequences.

Genes	Forward primers	Reverse primers
BSP	5′-TACCGAGCCTATGAAGATGA-3′	5′-CTTCCTGAGTTGAACTTCGA-3′
COL1	5′-GGAGGAGAGTCAGGAAGG-3′	5′-TCAGCAACACAGTTACACAA-3′
DMP-1	5′-GACTCTCAAGAAGACAGCAA-3′	5′-GACTCACTCACCACCTCT-3′
OCN	5′-TGAGTCCTGAGCAGCAG-3′	5′-TCTCTTCACTACCTCGCT-3′
OPN	5′-GTGGGAAGGACAGTTATGAA-3′	5′-CTGACTTTGGAAAGTTCCTG-3′
OSX	5′-TTGACATGTACCCCTTTCTG-3′	5′-CAATACCCCTGATGAAGAGG-3′
GAPDH	5′-GTATGACAACAGCCTCAAGAT-3′	5′-CCTTCCACGATACCAAAGTT-3′

## Data Availability

All the data in this study are available from the corresponding authors on reasonable request.

## Supplementary Materials

Supplementary materials Figure S1: verification of stem cell potentials in human dental pulp primary cells (hDPSCs). CD Antibodies against the following mesenchymal stem cell antigens were treated with cells: CD44, CD90, CD146, and STRO-1. CD24 and CD106 were used as negative markers. After incubation with the primary antibodies, FITC labeled anti-mouse secondary antibody was treated. CD expression of hDPSCs was analyzed by Flow cytometry. hDPSCs independently cultivated from six different patients were used in FACS analysis. Red peaks indicated cells treated with FITC-secondary antibody only. Figure S2: verification of odontogenic differentiation potential in human dental pulp primary cells (hDPSCs). Osteo/odontogenic gene expressions in hDPSCs treated with BMP-2 and BMP-4 for 2 weeks. hDPSCs independently cultivated from six different patients were used in Quantitative RT-PCR analysis. According to the data of relative expression between undifferentiated and differentiated cells, three cell batches isolated from patients #165, #280, and #311 were selected and used in this experiment.
