# Supplementary material for: 3D Spheroid Formation Using BMP-Loaded Microparticles Enhances Odontoblastic Differentiation of Human Dental Pulp Stem Cells
Source: Stem Cells Int. 2021 Aug 23;2021:9326298. doi: 10.1155/2021/9326298 (PMC8429013; doi:10.1155/2021/9326298)
Supplement: Supplementary materials — Figure S1: verification of stem cell potentials in human dental pulp primary cells (hDPSCs). CD Antibodies against the following mesenchymal stem cell antigens were treated with cells: CD44, CD90, CD146, and STRO-1. CD24 and CD106 were used as negative markers. After incubation with the primary antibodies, FITC labeled anti-mouse secondary antibody was treated. CD expression of hDPSCs was analyzed by Flow cytometry. hDPSCs independently cultivated from six different patients were used in FACS analysis. Red peaks indicated cells treated with FITC-secondary antibody only. Figure S2: verification of odontogenic differentiation potential in human dental pulp primary cells (hDPSCs). Osteo/odontogenic gene expressions in hDPSCs treated with BMP-2 and BMP-4 for 2 weeks. hDPSCs independently cultivated from six different patients were used in Quantitative RT-PCR analysis. According to the data of relative expression between undifferentiated and differentiated cells, three cell batches isolated from patients #165, #280, and #311 were selected and used in this experiment. [file 9326298.f1.docx]

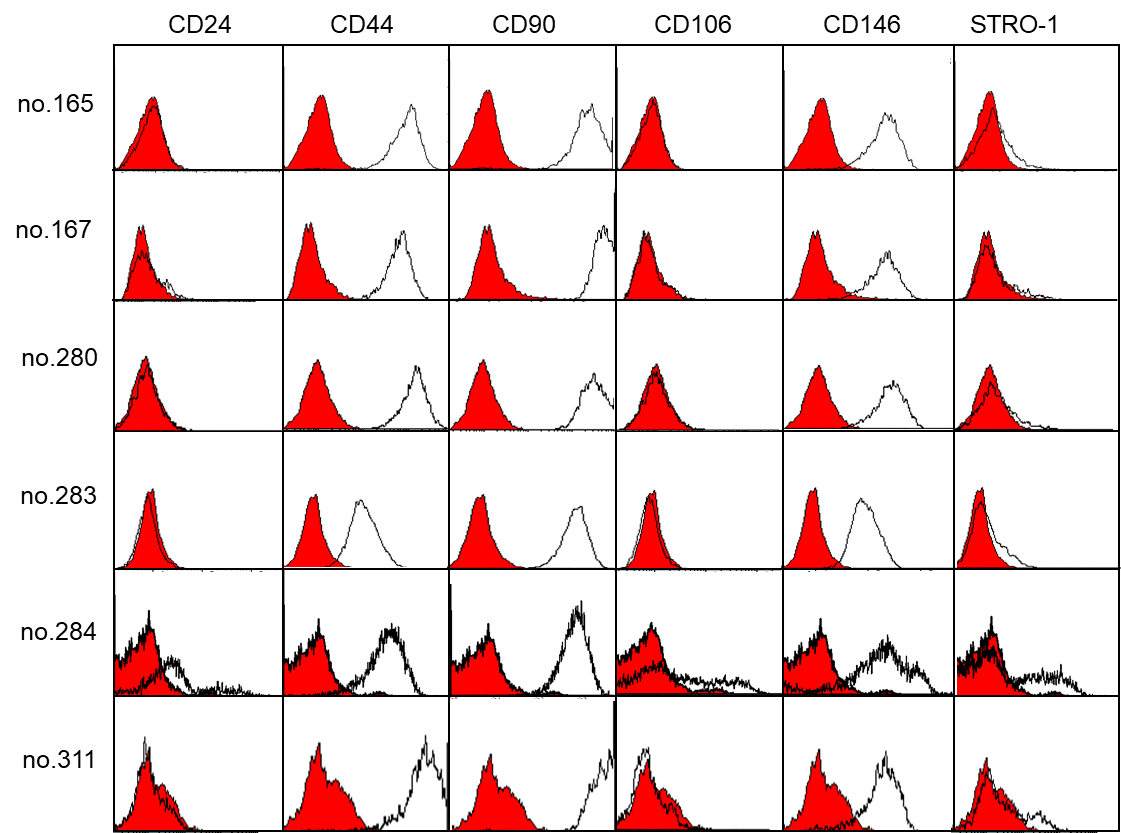


**Figure S1. Verification of stem cell potentials in human dental pulp primary cells (hDPSCs).** CD Antibodies against the following mesenchymal stem cell antigens were treated with cells: CD44, CD90, CD146, and STRO-1. CD24 and CD106 were used as negative markers. After incubation with the primary antibodies, FITC labeled ant-mouse secondary antibody was treated. CD expression of hDPSCs was analyzed by Flow cytometry. hDPSCs independently cultivated from six different patients were used in FACS analysis. Red peaks indicated cells treated with FITC-secondary antibody only.


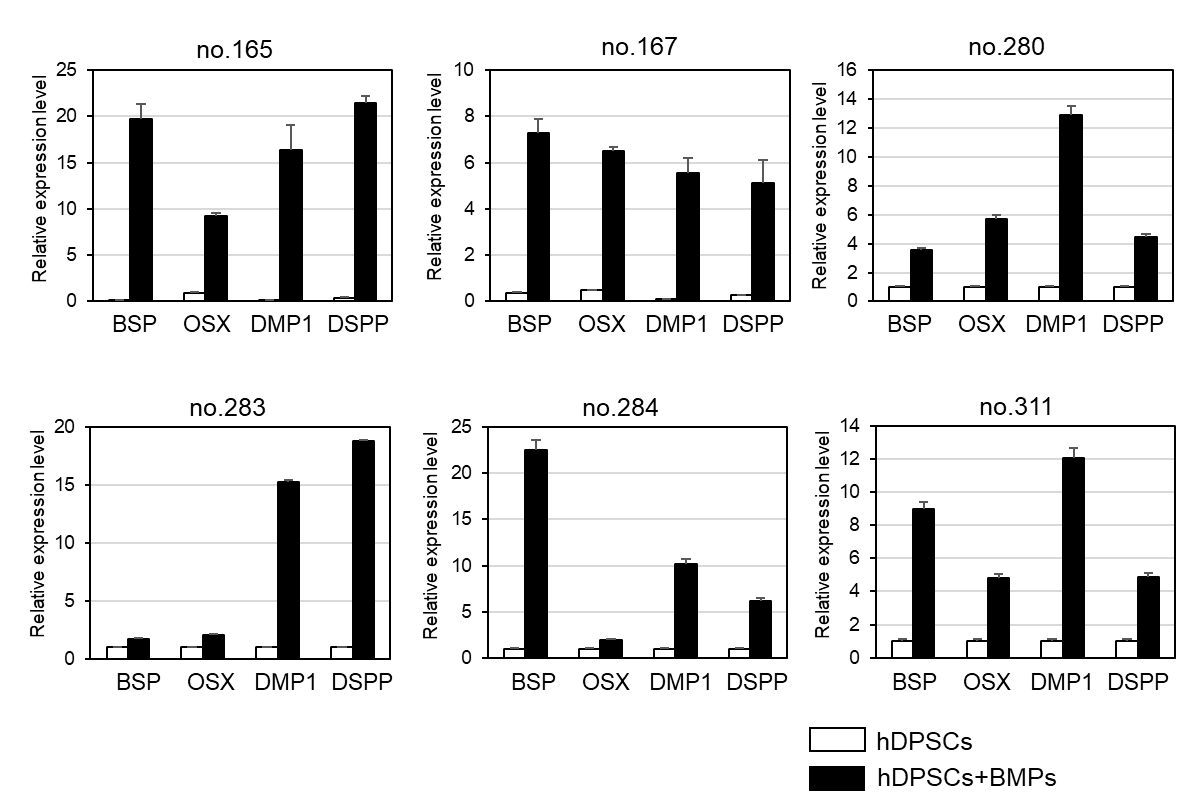


**Figure S2. Verification of odontogenic differentiation potential in human dental pulp primary cells (hDPSCs).** Osteo/odontogenic gene expressions in hDPSCs treated with BMP-2 and BMP-4 for 2 weeks. hDPSCs independently cultivated from six different patients were used in Quantitative RT-PCR analysis. According to the data of relative expression between undifferentiated and differentiated cells, three cell batches isolated from patient #165, #280, and #311 were selected and used in this experiment.
